# Case Report: Monocular Purtscher-like retinopathy secondary to systemic lupus erythematosus: analysis of clinical characteristics under low disease activity

**DOI:** 10.3389/fimmu.2026.1715542

**Published:** 2026-05-11

**Authors:** Xiaodong Li, Min Liu

**Affiliations:** Department of Ophthalmology, Guizhou University of Traditional Chinese Medicine, Guiyang, China

**Keywords:** systemic lupus erythematosus, Purtscher-like retinopathy, unilateral, case report, retinal microcirculation disorder

## Abstract

**Objective:**

This study aims to report a case of unilateral Purtscher-like retinopathy (PLR) secondary to systemic lupus erythematosus (SLE) and explore its clinical features, diagnosis, and therapeutic outcomes.

**Methods:**

A retrospective analysis was performed on the clinical data of a 23-year-old female SLE patient, including medical history, ophthalmic examinations, visual acuity, fundus assessment, optical coherence tomography (OCT), fundus fluorescein angiography (FFA), laboratory tests, and treatment follow-up results. The patient was admitted with acute left eye vision loss for over 2 days and had an 8-year history of SLE. Clinical manifestations included left eye Purtscher flecken and retinal microcirculatory abnormalities.

**Results:**

The initial left eye visual acuity was 0.05 (no improvement with correction). The fundus examination revealed Purtscher flecken along vascular tracts, OCT showed hyperreflectivity in the inner macular retina, and FFA confirmed PLR. The laboratory tests indicated elevated erythrocyte sedimentation rate (109.00 mm/h), increased immunoglobulin G (34.88 g/L), and a SLE Disease Activity Index score of 4 (low disease activity). The treatment included prednisone acetate combined with cyclophosphamide for primary disease control, Guhong Injection for circulatory improvement, peribulbar injection of compound anisodine combined with methylcobalamin for neuroprotection, and laser photocoagulation for macular non-perfusion areas. After 11 days of treatment, the left eye visual acuity improved to 0.25 with symptom relief; the 1-month follow-up showed reduced Purtscher flecken, improved macular hyperreflectivity, and stable vision.

**Conclusion:**

PLR is a rare ocular complication of SLE that can occur even during low disease activity. Early diagnosis combined with primary disease control, microcirculatory improvement, and laser therapy can effectively improve visual prognosis, highlighting the importance of regular fundus screening for SLE patients.

## Introduction

This report describes a rare instance of unilateral Purtscher-like retinopathy (PLR) occurring in a patient with systemic lupus erythematosus (SLE) during low disease activity (SLE Disease Activity Index (SLEDAI) score = 4). Unlike previous SLE-associated PLR cases, which typically present with bilateral involvement and high disease activity, our patient exhibited isolated left eye involvement without active systemic manifestations. This case underscores the importance of ophthalmic screening in SLE patients regardless of disease activity, as PLR may manifest even when systemic inflammation is quiescent.

## Case presentation

A 23-year-old female patient was admitted with “acute left eye vision loss and blurred vision for over 2 days.” Two days prior, she developed sudden left eye vision loss and blurred vision without an obvious precipitating factor, accompanied by left eye stabbing pain, photophobia, lacrimation, and ipsilateral headache. She was initially evaluated at another hospital, where macular OCT suggested left retinal artery occlusion (RAO). Despite symptomatic treatment, her condition did not improve, prompting referral to our institution for further management.

### Ophthalmic examination

In terms of visual acuity, the right eye (VOD) was 0.5 (corrected to 1.0) and the left eye (VOS) was 0.05 (no improvement with correction). The fundus findings include tortuous and dilated peripapillary vessels with numerous Purtscher flecken along the vascular tracts in the left eye. Intraocular pressure was at 13 mmHg bilaterally (1 mmHg = 0.133 Pa).

### Auxiliary examinations

**Figure 1:**
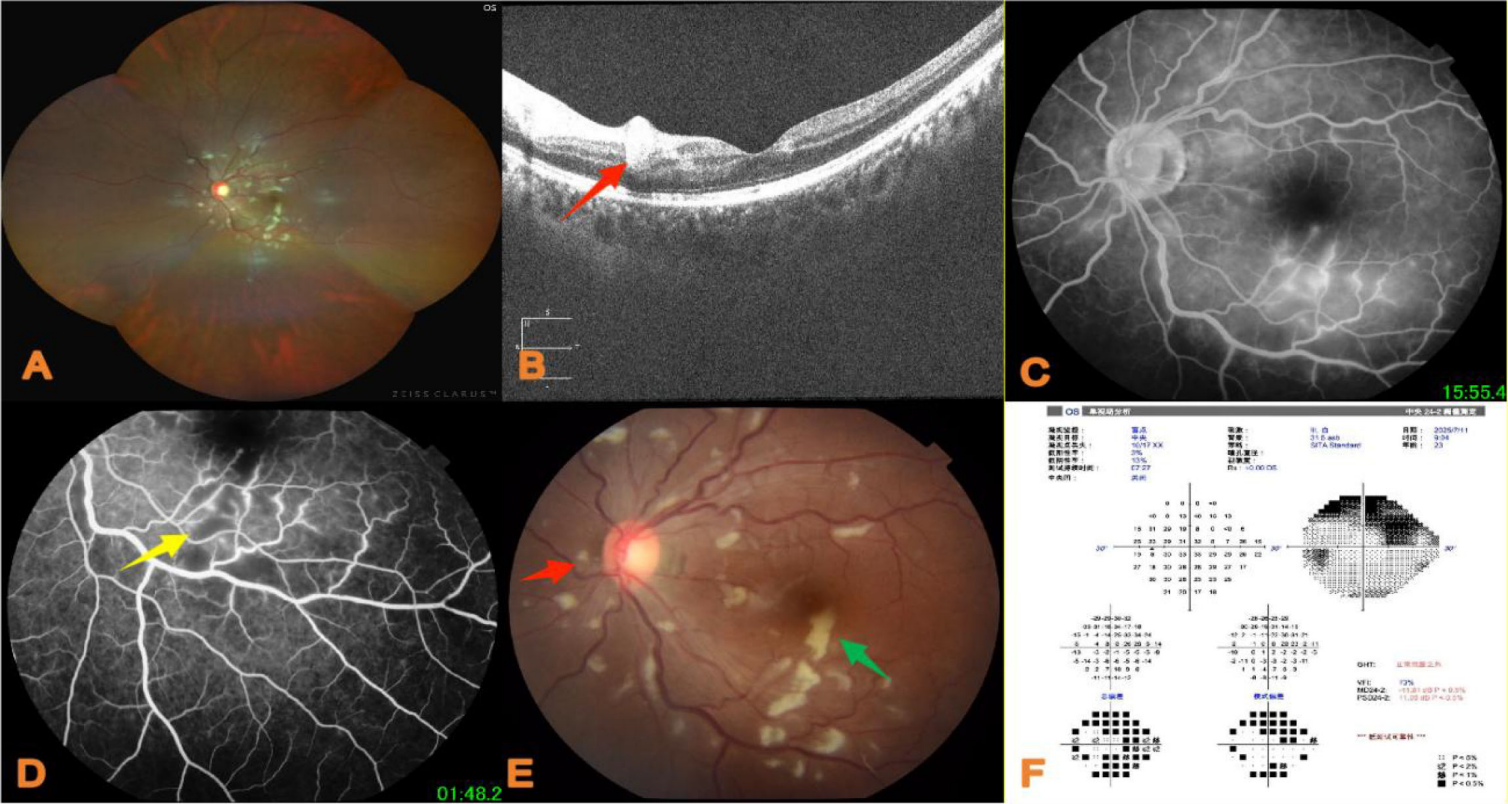
**(A)** At initial diagnosis, yellow-white Purtscher spots along the vascular course were observed in the left fundus. **(B)** OCT of the left macular area showing hyperreflection in the inner retinal layer (red arrow). **(C)** FFA of the left fundus revealing tortuous and dilated retinal vessels below the macular area, with early frosted-branch-like hyperfluorescence and obvious leakage in the late phase. **(D)** FFA of the left fundus showing small non-perfusion areas (yellow arrow). **(E)** Color fundus photograph of the left eye showing multiple large, pale cotton-wool spots, which were often confluent with ill-defined borders (red arrow, indicating Purtscher flecken), and scattered, isolated, small cotton-like cotton-wool spots with clear outlines (blue arrow) around the optic disc, which usually did not coalesce. **(F)** Visual field examination of the left eye showing a large visual field defect in the upper part.

In terms of fundus imaging, scanning laser ophthalmoscopy revealed tortuous, dilated retinal vessels and scattered Purtscher flecken in the left eye (**Figure 1A**). OCT showed hyperreflectivity in the inner retinal layers of the left macula (**Figure 1B**). For the fluorescein angiography: 20 mg of dye was taken orally once daily; cyclophosphamide at 0.4 g was advised for intravenous infusion every 2 weeks.

### Ocular and supportive therapy

Guhong injection: A daily intravenous infusion of 10 mL was given to improve circulation. A peribulbar injection of compound anisodine (2 mL) was also administered around the superficial temporal artery daily, combined with methylcobalamin tablets (0.5 g orally taken three times daily) for neurotrophic support. Local retinal laser photocoagulation was performed for macular non-perfusion areas (**Figure 1D**).

### Treatment outcomes

**Figure 2:**
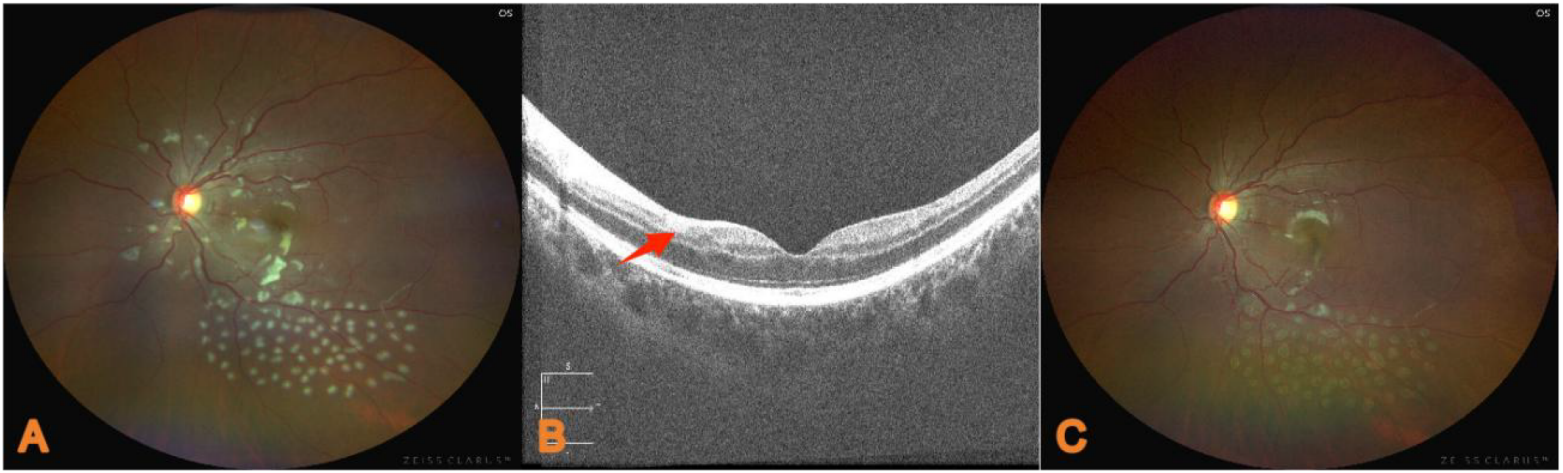
**(A)** During hospitalization, laser spots were observed in the retinal area below the macula of the left eye. **(B)** At the second consultation, OCT of the left macular area showed that the hyperreflective foci in the inner retinal layer were significantly reduced compared with the previous examination (red arrow). **(C)** Fundus examination of the left eye at the second consultation revealed the disappearance of Purtscher flecken in the optic disc and macular areas.

At 11 days post-treatment, the left eye visual acuity improved to 0.25. There was resolution of left eye stabbing pain, photophobia, lacrimation, and headache. At 1 month post-treatment, the fundus examination revealed laser spots inferior to the left macula (**Figure 2A**). OCT showed reduced hyperreflectivity in the inner retinal layers (**Figure 2B**). There was significant reduction in Purtscher flecken (**Figure 2C**). Visual acuity remained stable at 0.25.

## Discussion

SLE is a multisystem autoimmune disease characterized by hallmark malar butterfly rash, with additional clinical manifestations including joint pain, headache, and lymphadenopathy. It often requires differentiation from rheumatoid arthritis, mixed connective tissue disease, and Sjögren’s syndrome. Approximately one-third of the patients develop ocular symptoms (1). The pathogenesis of SLE-associated retinopathy is generally attributed to abnormal B-cell tolerance, which leads to immune complex formation and deposition in vascular walls, activating the complement system and triggering fibrinoid degeneration or necrosis of vessel walls. This vascular damage results in microvascular or macrovascular occlusion, ultimately causing retinal lesions. Clinically, cotton-wool spots are the most common manifestation (2), followed by retinal hemorrhage and vascular occlusion, consistent with the findings by Huang et al. (3). Notably, Purtscher flecken represents a special subtype of cotton-wool spots, typically presenting bilaterally and symmetrically; unilateral cases are rare. In this patient, characteristic PLR was observed in the left fundus (**Figure 1E**), potentially associated with occlusion of pre-capillary arterioles in the superficial and deep retinal layers (4). PLR is a non-traumatic retinopathy linked to systemic diseases, with a minimum reported incidence of 0.24 per million annually (5). Bilateral involvement is more common, accounting for approximately 60% of cases (6). However, some PLR cases have subtle clinical manifestations, suggesting that the actual incidence may be higher.

### Etiology and pathogenesis

PLR is primarily associated with non-traumatic factors such as SLE and acute pancreatitis. In Hashem’s study, SLE accounted for approximately 13.1% of PLR cases (7). The patient had a confirmed history of SLE and no major trauma, strongly implicating SLE as the etiology. While the exact pathogenesis of PLR remains incompletely understood, the prevailing hypothesis suggests that multiple triggers activate the complement system, inducing aggregation of leukocytes, platelets, and fibrin to form microemboli (8), which occlude pre-capillary retinal arterioles. In SLE, abnormal B-cell tolerance (caused by genetic, environmental, and other factors) leads to the activation of autoreactive B cells, which produce anti-double-stranded DNA antibodies. These antibodies form immune complexes with double-stranded DNA, which deposit on the walls of retinal blood vessels. The complexes activate the classical complement pathway, generating C3a and C5a. Both bind to receptors on endothelial cells, disrupting their tight junctions, inducing apoptosis, and chemotacting leukocyte aggregation. Leukocytes release substances that further damage blood vessels, forming an inflammatory cycle and ultimately triggering retinal vascular lesions. Among the above-mentioned mechanisms, abnormal B-cell tolerance is the “initiating factor”, immune complex deposition is the “key bridge”, complement activation is the “core amplifier”, and endothelial damage plus leukocyte aggregation is the “direct damage link”. These four elements together constitute the complete pathological chain of SLE-associated retinopathy (7). The core of the “leukocyte–platelet–fibrin microemboli” hypothesis is as follows: C3a and C5a generated by complement activation first chemotact and activate leukocytes, making them adhere to the vascular endothelium and release tissue factors to initiate the extrinsic coagulation pathway. Meanwhile, C3a and C5a activate platelets, enabling them to adhere to the damaged endothelium and release substances to recruit more platelets for aggregation. Subsequent coagulation cascade reactions promote the conversion of fibrinogen to fibrin; fibrin interweaves into a mesh, wrapping leukocytes and platelets to form microemboli. These microemboli move with the blood flow and eventually block the pre-capillary arterioles of the retina, leading to vascular occlusion (8). Concomitant factors—including fat emboli, vasospasm, sudden intracranial hypertension, and axoplasmic flow disturbances—synergistically impair retinal microcirculation. Vascular endothelial injury causes plasma leakage, and microthrombi extend to nerve fiber layer infarcts, forming Purtscher flecken. Finally, ischemia and exudation lead to visual field defects and vision loss.

### Clinical manifestations and diagnosis

PLR typically presents with sudden, painless bilateral or unilateral vision loss, often accompanied by visual field defects (9). Characteristic fundus findings include (1) Purtscher flecken—superficial, round, or irregular polygonal lesions in the posterior pole, indistinctly marginated, and distributed between retinal arterioles and venules; (2) cotton-wool spots—isolated, small, cotton-like white or yellow-gray patches around the optic disc with clear borders, usually non-confluent; (3) retinal hemorrhage—typically mild to moderate punctate or blot hemorrhages; and (4) edema—may involve the optic disc or macula (10). Literature reports indicate that OCT can detect cotton-wool spots in the retinal nerve fiber layer and Purtscher flecken in the inner nuclear layer. FFA reveals two types of abnormalities: ischemic changes (e.g., capillary non-perfusion, arteriolar occlusion) and leakage due to barrier disruption (e.g., vascular wall staining, late disc or retinal leakage, and choroidal fluorescence masking at exudative sites) (11). OCT angiography (OCTA) enables the early identification of retinal ischemic injury and precise localization of vascular occlusion, detecting subtle ischemia, microvascular occlusion, and infarction more sensitively than FFA (12). The widely accepted diagnostic criteria for PLR include (1) history of relevant systemic disease, (2) macular Purtscher flecken, (3) peripapillary cotton-wool spots, (4) disc or macular edema, (5) posterior pole flame-shaped or punctate hemorrhages, and (6) FFA evidence of capillary occlusion and non-perfusion areas. Diagnosis requires either criterion (1) or (2) plus any one of criteria (3)–(6) (13). In this case, the diagnosis of PLR is verified through a three-dimensional approach of “medical history + characteristic fundus manifestations + imaging evidence”, with specific basis as follows: underlying disease history—the patient has a 5-year history of SLE. Although the disease was in a low-activity phase at the time of this visit (SLEDAI score = 4), SLE is a clearly associated etiology of PLR, providing a foundational context for the diagnosis. In terms of characteristic fundus signs, the dilated fundus examination revealed scattered Purtscher flecken in the macular area (**Figure 1E**). These flecks, as a typical lesion of PLR, present as well-defined gray-white punctate lesions located in the retinal nerve fiber layer, which are specific manifestations of local infarction caused by microemboli. FFA evidence was established when the FFA examination showed retinal capillary non-perfusion areas (**Figure 1D**), mainly distributed around the macula and in the posterior pole. This is consistent with the hemodynamic changes in PLR caused by microembolic occlusion of pre-capillary arterioles. For OCT evidence, OCT demonstrated hyperreflective signals in the inner retinal layers (**Figure 1B**), corresponding to edema and microinfarction in the nerve fiber layer of the Purtscher flecken area, further confirming the pathological changes of PLR at the anatomical level.

### Differential diagnosis

Clinically, PLR must be differentiated from RAO and endophthalmitis (14). The patient was initially misdiagnosed with RAO at an outside hospital. RAO is often caused by cardiogenic or carotid embolism or atherosclerotic thrombosis, presenting with diffuse retinal gray edema and a “cherry-red spot” at the fovea. In contrast, PLR is characterized by multiple, peripapillary radially arranged Purtscher flecken (with or without hemorrhage), acute onset, severe vision loss, and absence of trauma—features that excluded RAO in this case. A summary of the key differential points between PLR, RAO, and hypertensive retinopathy is shown in **Table 1**.

**Table 1:** Summary of key differential points between PLR, RAO, and hypertensive retinopathy.

Differential points	PLR	RAO	Hypertensive retinopathy
Etiology	Systemic diseases; immune complex deposition activates complement, forming leukocyte-platelet microemboli occluding pre-capillary arterioles	Cardioembolic/carotid emboli, atherosclerotic thrombosis, causing central/branch retinal artery occlusion	Chronic hypertension-induced retinal arteriolar spasm and sclerosis; malignant hypertension triggers endothelial injury, exudation, and ischemia
Clinical manifestations	Acute unilateral/bilateral painless vision loss, possible visual field defects; often absent systemic hyperinflammation	Sudden painless severe vision loss, relative afferent pupillary defect; mostly unilateral, headache or carotid bruit	Asymptomatic in early stages; gradual vision decline in chronic phase; malignant hypertension presents with headache, nausea, and acute vision loss
Fundus examination	Characteristic Purtscher flecken; cotton-wool spots, minimal hemorrhage; no disc edema	“Cherry-red spot” diffuse retinal gray edema, arteriolar narrowing; pale optic disc	Arteriolar narrowing, arteriovenous nicking; grade hemorrhages, cotton-wool spots, hard exudates; grade optic disc edema
Auxiliary examinations	OCT: hyperreflectivity in inner retinal layers; FFA: capillary non-perfusion areas, vascular wall staining/leakage	OCT: retinal thickening/edema, loss of ellipsoid zone in macula; FFA: delayed or absent arterial filling, venous stasis	OCT: retinal nerve fiber layer thinning; FFA: microaneurysms, capillary dilation/leakage, non-perfusion in cotton-wool spot areas

### Treatment

Currently, there are no standardized guidelines or expert consensuses for the diagnosis and treatment of PLR. Clinical practice centers on the core principle of “treating the underlying systemic disease combined with ocular symptomatic therapy” to improve visual acuity, with treatment directions focusing on anti-inflammation, microcirculation improvement, hypoxia correction, and neuroprotection. For this PLR patient with comorbid SLE, each component of the combined treatment regimen (prednisone, cyclophosphamide, compound anisodine, methylcobalamin, Guhong injection, and laser photocoagulation) has clear justifications: prednisone and cyclophosphamide, as standard induction therapies for SLE-associated vasculitis, can inhibit inflammatory responses, complement activation, and immune complex deposition while stabilizing the blood-retinal barrier and neuronal cell membranes to promote nerve fiber repair (15, 16); compound anisodine (containing anisodine hydrobromide and procaine hydrochloride) improves the microcirculation by relieving the retinal arteriolar spasm and enhancing the choroidal blood flow (17); Guhong injection (each 10 mL contains 0.1 g aceglutamide and 50 mg *Carthamus tinctorius* extract, manufactured by Guizhou Bailing Group Pharmaceutical Co., Ltd., with NMPA approval number National Drug Approval H52020954) enhances retinal perfusion through the neuroprotective effect of aceglutamide and the antiplatelet, vasodilatory properties of *Carthamus tinctorius* extract. Domestic retrospective studies have shown that it can reduce the area of non-perfusion in PLR patients (17, 18), but this drug is only marketed in China and lacks validation from international multicenter randomized controlled trials (RCTs); methylcobalamin, a vitamin B12 derivative, can repair damaged retinal nerve cells, and multiple studies have confirmed its protective effect on retinal neuropathy (19, 20), and laser photocoagulation prevents neovascular complications by sealing non-perfusion areas. During treatment, SLE disease activity was monitored synchronously—every 2 weeks in the first month and monthly thereafter—using indicators such as anti-double-stranded DNA antibody titer, complement levels, and SLEDAI-2000 score. The results showed that the patient’s disease activity remained persistently low (score ≤2 at 1–3 months post-treatment), with no new systemic involvement.

However, the existing treatment regimen still has significant limitations in evidence: the efficacy data for compound anisodine and Guhong injection are mostly derived from small-sample domestic studies, lacking international validation. Additionally, Guhong injection has not been included in international guidelines for SLE or retinopathy, precluding head-to-head comparisons with internationally commonly used microcirculation drugs such as alprostadil. Although the combination of six treatment components achieves multi-target intervention, it is difficult to distinguish the specific efficacy contribution of a single drug, thus requiring further clarification through factorial-design RCTs. The current follow-up period of only 1 month is insufficient to fully evaluate long-term visual stability and PLR recurrence risk; subsequent follow-up should be extended to 6 months to 1 year to supplement the efficacy and safety data.

### Prognosis

PLR is prone to misdiagnosis, often delaying optimal treatment. Despite spontaneous resolution in some cases, visual prognosis is poor, with over 50% of patients experiencing poor final visual recovery (21). Disease progression can lead to severe complications, including retinal neovascularization, vitreous hemorrhage, expanded retinal ischemia, and worsening macular edema (22). The key clinical features of SLE-associated PLR cases are summarized in **Table 2**. In advanced stages, neovascular glaucoma may develop, potentially requiring enucleation (23). Poor prognostic factors include capillary or choroidal hypoperfusion and optic disc inflammation (23).

**Table 2:** Key clinical features of SLE-associated PLR cases.

Reference	Age/gender	SLE activity	PLR laterality	Treatment	Visual outcome
Kunavisarut et al. (21)	32/F	High	Bilateral	Corticosteroids	Poor (VA: 0.1)
Li et al. (22)	28/F	Moderate	Bilateral	Rituximab + IL-2	Improved (VA: 0.6)
Current case	23/F	Low (SLEDAI = 4)	Unilateral	Prednisone + laser photocoagulation	Improved (VA: 0.25)

## Conclusion

PLR is a rare ocular complication of SLE, and its occurrence may not align with systemic disease activity—for instance, this patient had a SLEDAI score of 4 (low activity) but developed sudden vision loss and visual field defects, indicating that PLR can occur even with stable systemic disease. Thus, regular fundus screening is crucial for all SLE patients. Clinicians should consider PLR in the differential diagnosis of unexplained monocular acute vision loss to avoid misdiagnosis or missed diagnosis.

## Data Availability

The raw data supporting the conclusions of this article will be made available by the authors, without undue reservation.
